# Supplementary material for: Seed Dormancy and Seedling Ecophysiology Reveal the Ecological Amplitude of the Threatened Endemism Picris willkommii (Schultz Bip.) Nyman (Asteraceae)
Source: Plants (Basel). 2022 Jul 29;11(15):1981. doi: 10.3390/plants11151981 (PMC9370197; doi:10.3390/plants11151981)
Supplement: Supplementary file 1 [file plants-11-01981-s001.zip › plants-1808306-supplementary.pdf]

Article

Seed dormancy and seedling ecophysiology reveal the ecological amplitude of the threatened endemism *Picris willkommii* (Schultz Bip.) Nyman (Asteraceae)

Manuel Fernández * and Raúl Tapias

Department of Agroforestry Sciences, School of Engineering, University of Huelva, Avda. Fuerzas Armadas, s/n, 21071 Huelva, Spain; rtapias@dcaf.uhu.es (R.T.)

* Correspondence: manuel.fernandez@dcaf.uhu.es (M.F.); Tel.: +34-959-217-712; Fax: +34-9592-17560

Supplementary material

Table S1. Climatic conditions in the study área. Weather station "El Moral" (Ayamonte, Spain): UTM, zone 29S, X: 647266, Y: 4117116, 2 m.a.s.l. Time period: 18 years.

**Month**	**P** (mm)	**T** (°C)	**TMax** (°C)	**TMin** (°C)	**TM** (°C)	**Tm** (°C)
January	70.9	10.7	19.5	-1.0	14.7	6.8
February	49.6	12.3	25.0	1.5	16.3	8.2
March	33.2	14.6	27.5	2.0	19.3	9.9
April	43.2	15.5	29.5	4.0	20.1	10.8
May	28.1	18.3	31.5	8.0	22.8	13.8
Jun	7.0	21.2	37.0	10.0	25.9	16.6
July	3.4	24.6	37.0	10.0	29.9	19.3
August	2.7	24.1	39.0	14.0	29.2	19.1
September	11.2	22.2	36.0	10.0	27.0	17.4
October	60.6	18.4	33.0	7.5	22.6	14.2
November	91.8	14.9	26.0	3.0	18.9	10.8
December	121.7	12.6	23.0	1.5	16.2	9.0
**Mean annual**	523.4	17.4				

P: mean precipitation, T: mean temperature, TMax: absolute maximum temperature, TMin: absolute minimum temperature, TM: mean daily maximum temperatures, Tm: mean daily minimum temperatures.

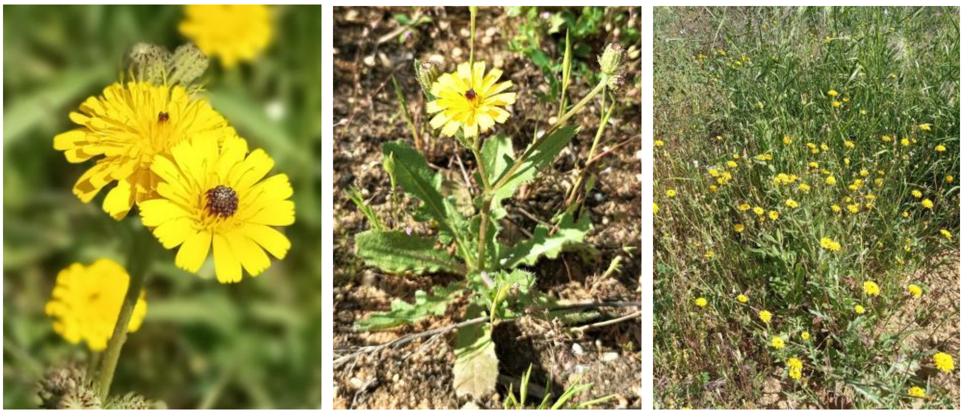

Figure S1. (**Left**) flowers of *Picris willkommii* showing the typical dark color of the central ligules when the flower is not fully developed; (**Center**) a small *Picris willkommii* plant; (**Right**) *Picris willkommii* plants among the grassland.

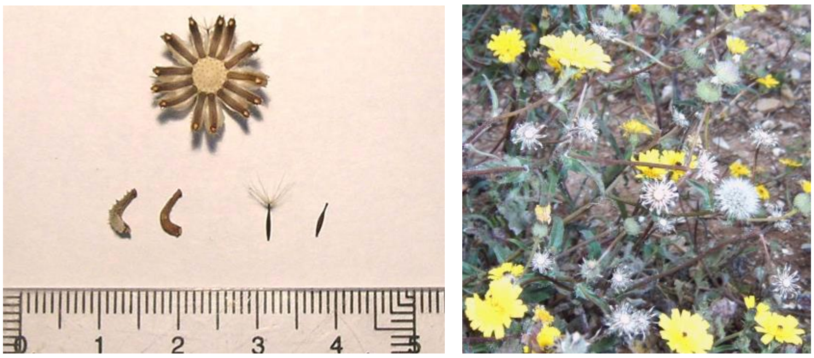

Figure S2. (**Left**) *Picris willkommii* receptacle after dispersal of the central achenes, with peripheral achenes still attached; two peripheral achenes on the left and two central ones on the right, one of them still retains the plumose pappus. (**Right**) *Picris willkommii* plants showing at the same time developing flowers, receptacles with plumose achenes, and receptacles where the central achenes have already dispersed.

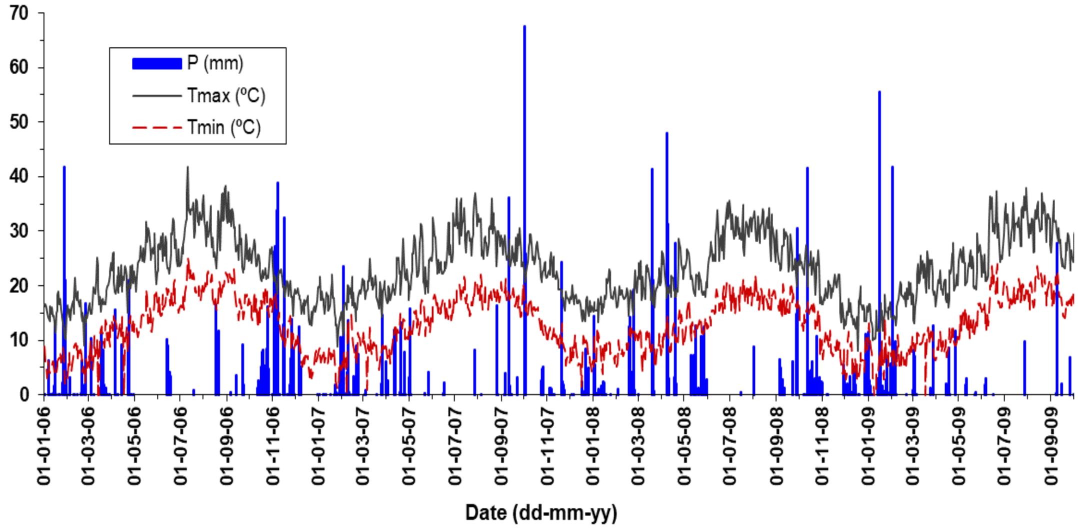

Figure S3. Seasonal pattern of daily maximum temperatures (Tmax), daily minimum temperatures (Tmin) and precipitation (P) from January 2006 to September 2009 at the study area. Weather station ("Lepe", Spain): UTM, zone 29S, X: 655 814, Y: 4,123,035, 74 m.a.s.l.

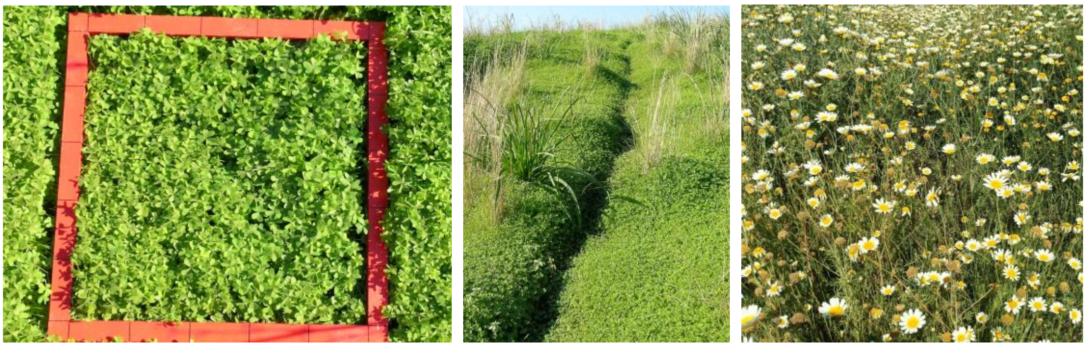

Figure S4. Examples of areas within the natural range of *Picris willkommii* where the species composition of the natural grassland has been altered and replaced by a single dominant species such as *Oxalis pes-caprae* L. (**Left** and **Center***)* and *Chrysanthemum coronarium* L. (**Right**).

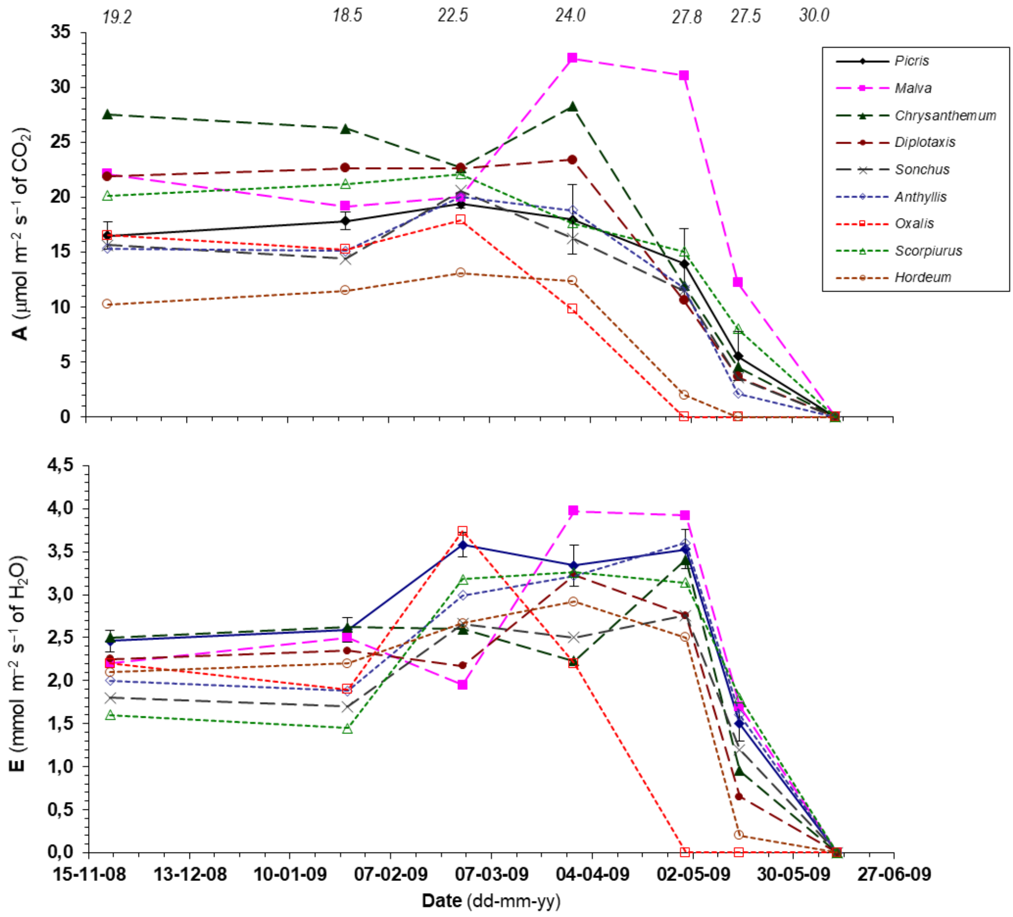

Figure S5. Seasonal variation of average net photosynthetic (A) and transpiration (E) rates for nine plant species on sunny days. The numbers above indicate the ambient temperature at the measurement time (°C). Species: *Picris willkommii* (Schultz Bip.) Nyman, *Malva sylvestris* L., *Chrysanthemum coronarium* L., *Diplotaxis virgata* (Cav.) DC., *Sonchus asper* (L.) Hill, *Anthyllis cytisoides* L., *Oxalis pes-caprae* L., *Scorpiurus sulcatus* L. and *Hordeum murinum* L. In order to clarify the figure, only the error bars corresponding to *Picris willkommii* have been represented.

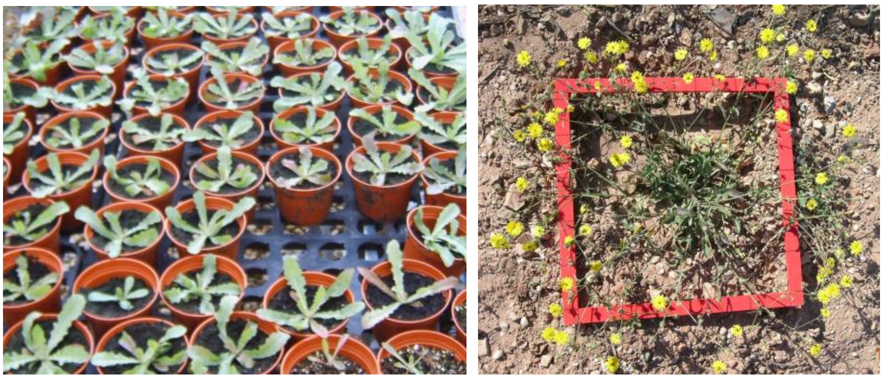

Figure S6. (**Left**) *Picris willkommii* seedlings grown in pots in the nursery used for the cold tolerance tests and for the field planting trials. (**Right**) a *Picris willkommii* plant in the field 2.5 months after being transplanted from the nursery.

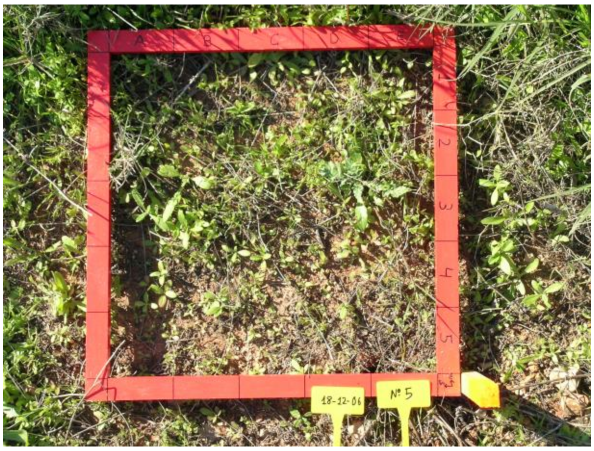

Figure S7. A sampling plot (0.5 m × 0.5 m) with divisions every 10 cm to facilitate counting, and the stake in the NE corner in order to locate the plot at the same site on all sampling dates.

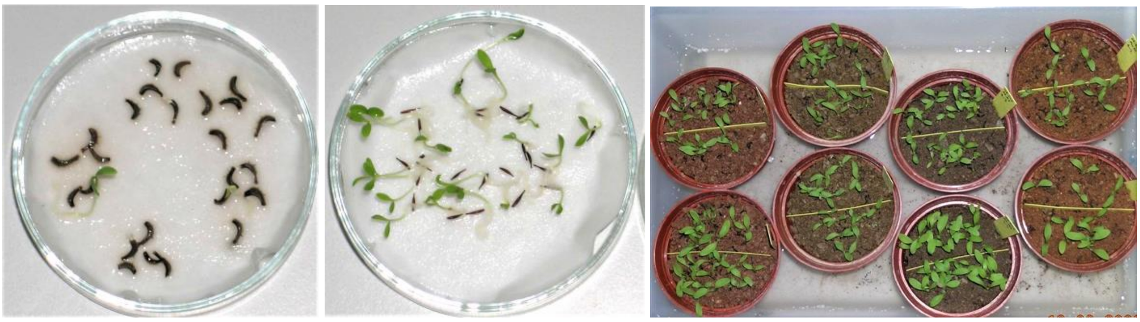

Figure S8. *Picris willkommii* seeds in the germination tests. (**Left**) peripheral seeds in a Petri dish on moistened filter paper; (**Center**) central seeds in a Petri dish on moistened filter paper; (**Right**) peripheral seeds germinating in pots on natural substrates.
